# Purification and Characterization of a Polyextremophilic ***α***-Amylase from an Obligate Halophilic *Aspergillus penicillioides* Isolate and Its Potential for Souse with Detergents

**DOI:** 10.1155/2015/245649

**Published:** 2015-06-09

**Authors:** Imran Ali, Ali Akbar, Mohammad Anwar, Sehanat Prasongsuk, Pongtharin Lotrakul, Hunsa Punnapayak

**Affiliations:** ^1^Plant Biomass Utilization Research Unit, Department of Botany, Faculty of Science, Chulalongkorn University, Bangkok 10330, Thailand; ^2^Institute of Biochemistry, University of Balochistan, Quetta 87300, Pakistan; ^3^Department of Microbiology, University of Balochistan, Quetta 87300, Pakistan

## Abstract

An extracellular *α*-amylase from the obligate halophilic *Aspergillus penicillioides* TISTR3639 strain was produced and enriched to apparent homogeneity by ammonium sulfate precipitation and Sephadex G100 gel filtration column chromatography. The mass of the purified amylase was estimated to be 42 kDa by SDS-PAGE. With soluble starch as the substrate it had a specific activity of 118.42 U·mg^−1^ and *V*_max⁡_ and *K*_*m*_ values of 1.05 *µ*mol·min^−1^·mg^−1^ and 5.41 mg·mL^−1^, respectively. The enzyme was found to have certain polyextremophilic characteristics, with an optimum activity at pH 9, 80°C, and 300 g·L^−1^ NaCl. The addition of CaCl_2_ at 2 mM was found to slightly enhance the amylase activity, while ZnCl_2_, FeCl_2_, or EDTA at 2 mM was strongly or moderately inhibitory, respectively, suggesting the requirement for a (non-Fe^2+^ or Zn^2+^) divalent cation. The enzyme retained more than 80% of its activity when incubated with three different laundry detergents and had a better performance compared to a commercial amylase and three detergents in the presence of increasing NaCl concentrations up to 300 g·L^−1^. Accordingly, it has a good potential for use as an *α*-amylase in a low water activity (high salt concentration) and at high pH and temperatures.

## 1. Introduction

Hypersaline environments are caused by the evaporation of water and they are also called thallasohaline environment. Due to the evaporation process the sodium chloride (NaCl) concentration rises above 300 psu. Many microbial communities have been found from the sesalterns. Halophilic microorganisms are extremophiles that are able to survive in and may require salt for their growth [1]. Fungi that are isolated from hypersaline environments with a salinity above 100 g·L^−1^ and are able to grow* in vitro* at a 175 g·L^−1^ salt concentration are categorized as halophilic fungi [2, 3]. Very few fungi have been reported yet to inhabit the hypersaline habitats. Their function in these environments is still not fully understood [2]. Unlike other microbes, the fungi can grow independent of salt concentration in saline environments [1]. However, obligate halophilic fungi are those that are unable to grow in the absence of a salt concentration [4].

Extremophilic microorganisms adopt different strategies to survive in extreme available conditions. They harbor different metabolites such as enzymes that can work at extreme conditions [5] and so are of interest for diverse biotechnological applications. Halophiles are currently used in several fermentation processes [6], such as for the production of bioactive compounds [7], biorhodopsin, biosurfactants, food additives, and biocompatible solutes [8]. Extreme halophiles have been increasingly investigated for their hydrolytic enzymes since these have potential uses in several industrial applications [9, 10]. However, the use of halophilic microorganisms and their metabolites has largely involved halophilic bacteria [11]. Despite the fact that halophilic fungi, especially the obligate strains, are better sources of extracellular enzymes, they have not been investigated very much for their potential in biotechnological applications [3, 12]. *α*-Amylase (1,4-*α*-D-glucan glucanohydrolase, EC 3.2.1.1) is a class of important industrial enzymes that are used in the food, textile, laundry, and pharmaceutical industries [13, 14] and currently form about 25% of the total enzyme market [15]. Although many microbial amylases have been used as an additive in laundry detergents [16], they do not perform well in hard or saline water, which then limits their use in such areas [3, 12].

Recently, the obligate halophilic* Aspergillus penicillioides* TISTR 3639 strain was isolated from an extreme hypersaline environment (a man-made solar saltern) in the Ban Laem district of Phetchaburi province, Thailand [1, 4]. The fungus was found to be positive for extracellular *α*-amylase activity [3] by plate screening method (Supplementary Figure S1; see Supplementary Material available online at http://dx.doi.org/10.1155/2015/245649). In this study, the purification and characterization of *α*-amylase from* A. penicillioides* TISTR 3639 were performed. Due to its polyextremophilic properties, the potential of using this enzyme as a laundry detergent additive was then investigated.

## 2. Materials and Methods

### 2.1. Growth Conditions for Enzyme Production

The* A. penicillioides* TISTR3639 strain was grown in 150 mL flasks containing 100 mL of production medium (PM) at room temperature (25 ± 2°C) at 150 rpm for 14 d. The PM was made according to Ali et al. [3] with a few modifications such that the composition was composed of 10 g·L^−1^ soluble starch, 3.0 g·L^−1^ mycological peptone, 100 g·L^−1^ NaCl, 8.0 g·L^−1^ CaCO_3_, 6.6 g·L^−1^ (NH_4_)_2_SO_4_, 3.5 g·L^−1^ KH_2_PO_4_, 0.15 g·L^−1^ FeSO_4_·7H_2_O, and 0.10 g·L^−1^ MgSO_4_·7H_2_O.

### 2.2. Amylase Purification

Enrichment to apparent homogeneity of the *α*-amylase was performed by ammonium sulfate precipitation and Sephadex G100 gel filtration chromatography as previously described [12, 17, 18]. The 14 d grown culture broth (100 mL) was centrifuged at 13,000 ×g at 4°C for 10 min and the supernatant was harvested. The amylase was then precipitated by bringing the supernatant to 90% saturation (NH_4_)_2_SO_4_, storing overnight at 4°C, and harvesting the insoluble fraction by centrifugation at 12,000 ×g for 30 min. The pellet was then dissolved in 100 mM Tris-HCl buffer (pH 8) and dialyzed against the same buffer for 48 h. The dialyzate was then subjected to Sephadex G100 gel filtration using a 2.6 cm × 150 cm column, preequilibrated in and then eluted with 25 mM Tris-HCl buffer (pH 8) containing 5 mL·L^−1^ Triton X-100 at a flow rate of 30 mL·h^−1^. Fractions (5 mL) were collected and each was tested for *α*-amylase activity and total protein content.

Determination of the purity and molecular weight of the enriched *α*-amylase was performed using sodium dodecyl sulphate polyacrylamide gel electrophoresis (SDS-PAGE) resolution (150 g·L^−1^ resolving gel) followed by coomassie blue staining, as reported by Hmidet et al. [18]. The purified amylase was mixed at a 1 : 5 volume ratio with the loading buffer (10 mM Tris-HCl, pH 8, 25 g·L^−1^ SDS, 50 mL·L^−1^ *β*-mercaptoethanol, 10 mL·L^−1^ glycerol, and 0.002 g·L^−1^ bromophenol blue). Prior to loading and electrophoresis, the sample was denatured and reduced by heating at 100°C for 5 min. Gels were stained with 2.5 g·L^−1^ Coomassie Brilliant Blue R250 in 450 mL·L^−1^ ethanol-100 mL·L^−1^ acetic acid and destained with 50 mL·L^−1^ ethanol-7.5 mL·L^−1^ acetic acid. The molecular weight was determined in comparison to the Unstained Precision Plus Protein 161 molecular marker kit (Bio-Rad, USA).

### 2.3. *α*-Amylase Assay

*α*-Amylase activity was determined by the 3,5-dinitrosalicylic acid (DNS) method as described by Miller [19] using 10 g·L^−1^ soluble starch as the substrate. The reaction mixture (0.1 mL enzyme solution, 0.5 mL 0.1 M phosphate buffer, and 5 mg soluble starch) was incubated at 40°C for 10 min. The reaction was then stopped by the addition of 3 mL of DNS and heating in a boiling water bath for 5 min. After cooling, 10 mL of water was added and the absorbance of the reaction mixture was read at 540 nm (A_540_). One unit (U) of enzyme activity was defined as the amount of enzyme that produced 1 *μ*mol of glucose in 1 min.

### 2.4. Protein Estimation

The amount of protein was estimated by the method of Lowry [20], using bovine serum albumin (BSA) as the standard.

### 2.5. Characterization of the Enriched *α*-Amylase

The effect of the pH, temperature, and NaCl concentration on the enriched amylase enzyme activity was evaluated by sequential univariate analysis of the pH, temperature, and NaCl concentration, respectively, and monitoring the relative enzyme activity (the highest activity was referred to as 100%) as the selected parameter. For evaluation of the optimal pH, the reaction mixture was incubated in 0.1 M acetate buffer for pH 5-6 and 0.1 M phosphate buffer for pH 7–12, at a constant 30°C with no added NaCl. For evaluation of the optimal temperature, the reaction mixture was incubated at a temperature range of 40–100°C in 0.1 M phosphate buffer at the optimal pH (as determined above). Finally, for evaluation of the optimal salinity level, the sample mixture in 0.1 M phosphate buffer at the found optimal pH was supplemented with NaCl to a final concentration of 0–500 g·L^−1^ and incubated at the optimal temperature.

The effect of various metal ions, or the enzyme inhibitors *β*-mercaptoethanol and EDTA, on the enzyme activity was investigated by separately adding BaCl_2_, CaCl_2_, FeCl_2_, HgCl_2_, MgCl_2_, and ZnCl_2_ to the reaction mixture at a final concentration of 2 mM. The relative amylase activity (%) was calculated in comparison to that without any additives.

### 2.6. Determination of the Kinetic Parameters of the Enriched *α*-Amylase

The kinetic parameters of the enzyme were determined by incubating the enriched *α*-amylase with 0.1–40 g·L^−1^ soluble starch (substrate) under the previously found optimum conditions. The *V*_max⁡_ and *K*_*m*_ values were then calculated from the Lineweaver-Burk plot.

### 2.7. Compatibility of the Enriched *α*-Amylase with Commercial Detergents

The compatibility of the enriched *α*-amylase with three commercial detergents (a liquid detergent (A) and two powdered detergents (B and C) bought from Talad Thai market, Pathumthani, Thailand) was determined in terms of the respective enzyme activities in comparison with that of the enriched enzyme in the absence of the detergents.

The detergent solutions were prepared by dissolving the powdered or liquid detergent in distilled water to 7 g·L^−1^ and heating at 100°C for 90 min to denature any enzyme activity present in the solutions. The cooled solutions were then mixed with the purified amylase at a 1 : 1 volume ratio and incubated for 1 h at 40°C. The residual enzyme activity was calculated in comparison with the control (distilled water instead of the detergents solution), expressed as the relative activity (%) of the control experiment.

### 2.8. Performance of the Enriched *α*-Amylase with Commercial Detergents in Varying NaCl Concentrations

For the performance test, the control mixture was made using 0.1 mL of the enriched *α*-amylase from* A. penicillioides* TISTR3639 in 0.5 mL of 0.1 M phosphate buffer (pH 7) containing 10 g·L^−1^ of soluble starch and 0–50 g·L^−1^ NaCl, while the three detergents (A, B, and C) were assayed as above except for adding the respective detergent instead of the enriched amylase. A commercial *α*-amylase from* A. oryzae* (Sigma Aldrich, Germany) was also used for comparison.

### 2.9. Statistical Analysis

Each experiment with the required controls was performed in triplicate and the data are presented as the mean ± one standard deviation (SD). Significance of the differences between means was tested for by analysis of variance (ANOVA) and Duncan's multiple means tests (DMMT) on the parametric or arc-sine square root transformed data using the SPSS software, where a *P* value of less than 0.05 was considered as significant.

## 3. Results

### 3.1. Enrichment of the *α*-Amylase

**Figure 1:**
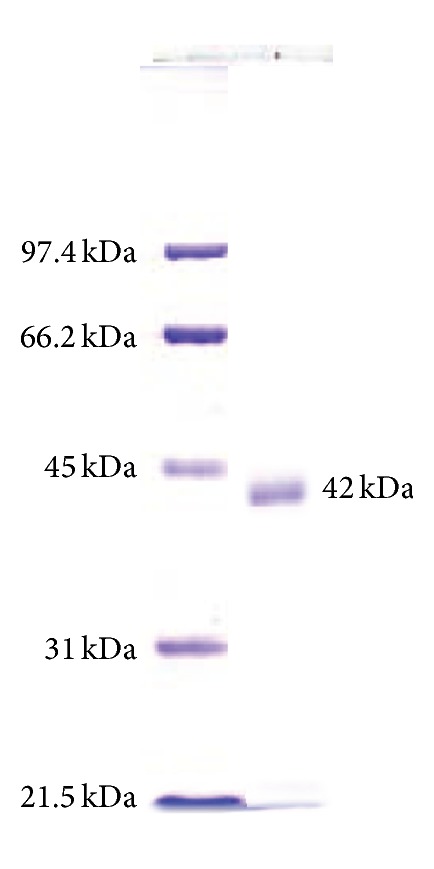
SDS-PAGE analysis of the enriched *α*-amylase from* A. penicillioides* TISTR 3639. Lane 1: molecular mass ladder; lane 2 the enriched *α*-amylase, showing a single band at approximately 42 kDa.

The initial 90% saturation (NH_4_)_2_SO_4_ cut gave a 2.5-fold increased enzyme specific activity for a 32% yield loss and a 76% total protein reduction (Table 1). Following Sephadex G100 gel filtration, the eluted fraction showing the highest amylase activity was then evaluated for its apparent purity and molecular weight by SDS-PAGE resolution and coomassie blue staining. Only a single protein band was visualized suggesting the apparent enrichment of the enzyme to homogeneity (Figure 1). Overall, the enzyme activity was increased 6.96-fold following the 90% saturation (NH_4_)_2_SO_4_ precipitation and Sephadex G100 gel filtration for a loss of 56% activity. The specific activity of the enriched *α*-amylase was estimated to be 118.4 ± 1.2 U·mg^−1^ (Table 1), with an estimated molecular weight of ~42 kDa by SDS-PAGE analysis (Figure 1).

### 3.2. Characterization of the Enriched *α*-Amylase

**Figure 2:**
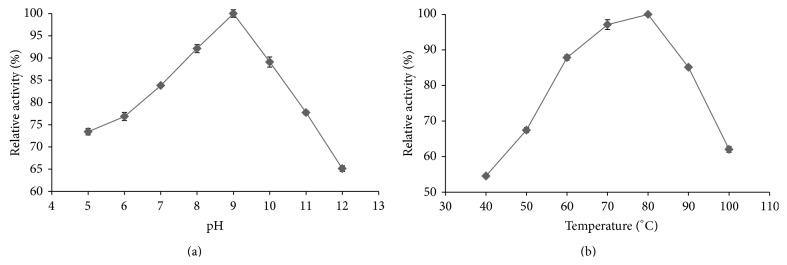
Effect of the (a) pH (at 30°C) and (b) temperature (at pH 9.0) on the activity of the enriched *α*-amylase from* A. penicillioides* TISTR 3639. Data are shown as the mean relative activity (%) ±1 SD (error bars), derived from three repeats. Means with a different lowercase superscript letter (a, b, and c) are significantly different (ANOVA and DMRT of the transformed data, *P* < 0.05).

The effect of increasing the pH (range 5–12) on the enriched *α*-amylase activity, when assayed at 30°C, is shown in Figure 2(a), where the enzyme activity was higher (>80%) in a neutral to moderately alkaline pH (7–10). The enzyme activity declined above and below pH 9, but this decline was more marked with increasing pH above 9 (64% activity at pH 12) than with decreasing pH (~75 and ~73% at pH 6 and 5, resp.).

Increasing the temperature increased the activity of the enriched *α*-amylase, as assayed at the more optimal pH 9 (Figure 2(b)), to peak at 80°C (100 ± 0.81%), and then declined at temperatures above 80°C, but it still retained more than 60% of its initial activity at 100°C. Thus, the thermophilic nature of the enzyme at temperatures higher than 60°C was clearly revealed.

**Figure 3:**
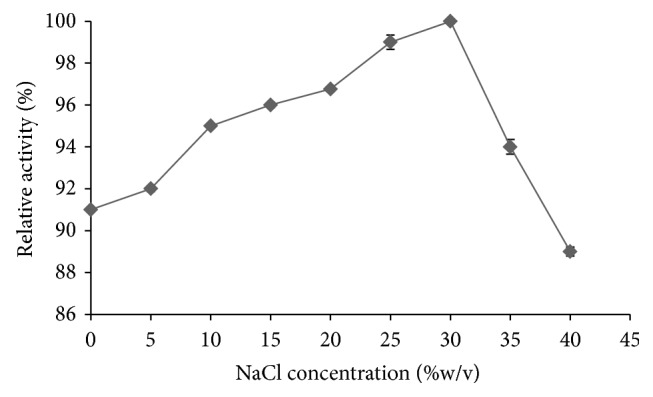
Effect of the NaCl concentration (g·L^−1^) on the activity of the enriched *α*-amylase from* A. penicillioides* TISTR 3639 at pH 9 and 80°C. Data are shown as the mean relative activity (%) ±1 SD (error bars), derived from three repeats. Means with a different lowercase superscript letter (a, b, and c) are significantly different (ANOVA and DMRT of the transformed data, *P* < 0.05).

With respect to the effect of the salinity, a gradual increase in the enriched *α*-amylase enzyme activity was observed with increasing NaCl concentrations up to 300 g·L^−1^, as assayed at pH 9 and 80°C (Figure 3). Although there was a sharp decrease in the enzyme activity above 300 g·L^−1^ NaCl, the enzyme was still able to retain most (89 ± 0.21%) of its initial activity at the nearly saturated salt concentration of 400 g·L^−1^ NaCl.

Finally, the effect of various metal ions and enzyme inhibitors at a concentration of 2 mM on the activity of the enriched *α*-amylase is summarized in Table 2. Except for CaCl_2_, which only weakly (but statistically significantly) increased the activity, none of the metal ions and enzyme inhibitors at this concentration were found to increase the enzyme activity. The slight decrease in the enzyme activity with the addition of 2 mM BaCl_2_, HgCl_2_, and* b*-mercaptoethanol was significant all the same, whilst the enzyme was moderately inhibited by the inclusion of FeCl_2_ or EDTA and strongly inhibited by ZnCl_2_.

### 3.3. Enzyme Kinetics

**Figure 4:**
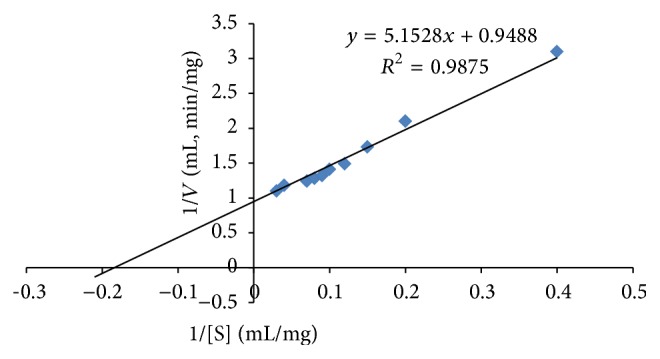
Lineweaver-Burk plot for the determination of the *V*_max⁡_ and *K*_*m*_ values of the enriched *α*-amylase from* A. penicillioides* TISTR 3639, at optimum conditions (pH 9 and 80°C), in the presence of different concentrations of soluble starch.

From the Lineweaver-Burk plot (Figure 4), the enriched amylase had a *K*_*m*_ of 5.41 mg·mL^−1^ and *V*_max⁡_ of 1.05 *μ*mol·min^−1^·mg^−1^ with soluble starch as the substrate.

### 3.4. Compatibility of the Enriched *α*-Amylase with the Selected Three Commercial Detergents

The compatibility results of the enriched enzyme with three commercial detergents (A, B, and C) as well as the commercial amylase enzyme are summarized in Table 3. The residual activity taken in percentage, as compared with control, showed that the enzyme was seen to retain more than 80% of the activity without any added detergent. The powdered B and C detergents were found to be more compatible with the enriched amylase, by giving the residual enzyme activity of 87% and 94%, respectively, than was liquid detergent A, which was observed to provide 81% amylase residual activity.

### 3.5. Performance Test with Commercial Detergents

**Figure 5:**
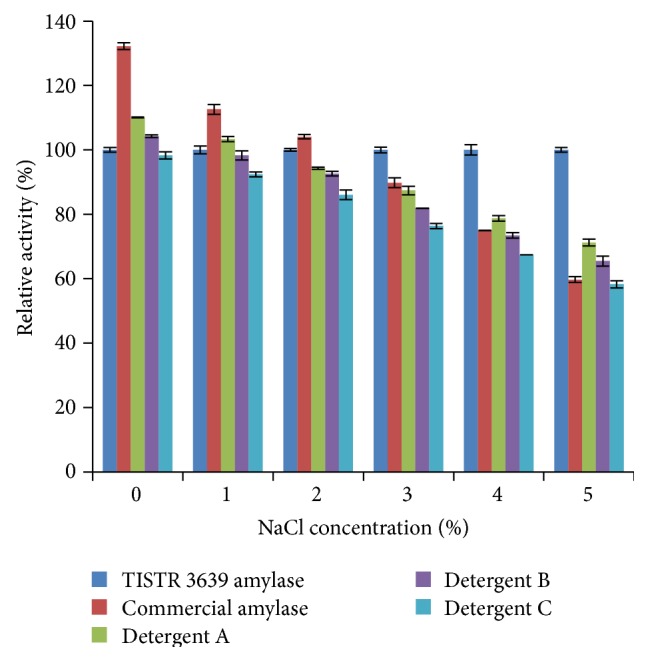
Performance comparison of a commercial amylase, detergents A, B, and C, and the enriched *α*-amylase (control) from* A. penicillioides* TISTR 3639 at pH 7 and 30°C in the presence of different concentrations of NaCl. The results are expressed as the relative activity (%) to that of the enriched *α*-amylase from* A. penicillioides* TISTR 3639 without NaCl and are shown as the mean relative activity (%) ±1 SD (error bars), derived from three repeats. Means with a different lowercase superscript letter (a, b, and c) are significantly different (ANOVA and DMRT of the transformed data, *P* < 0.05).

The performance test results of this enriched *α*-amylase in the presence of the respective commercial detergents are summarized in Figure 5, where the relative activity (%) was found to decrease with increasing salt concentration. However, in similar salt conditions the commercial amylase and the detergents had an inferior activity in comparison to this enriched *α*-amylase from* A. penicillioides*. At 50 g·L^−1^ NaCl, only 60% of the* A. penicillioides* amylase activity was detected in the commercial amylase and 60–70% in the three detergents.

## 4. Discussion

Many attempts have been made to find suitable fungus strains for the production of amylases with desirable properties [21]. Mesophilic fungi are reported to be the principal amylase producers [16] and especially members of the* Aspergillus* and* Penicillium* genera that appear to be the dominant producing species [22]. Fungal amylases are preferred for use in various industries, including the food and pharmaceutical industries, due to their nontoxic characteristics [3, 12, 23]. Consequentially,* Aspergillus* species, such as* Aspergillus niger* and* Aspergillus oryzae*, are frequently used in the industrial production of amylases [24], but there are few reports on the purification and detailed characterization of *α*-amylases from halophilic fungi [12]. In addition, to the best of our knowledge, this is the first report of the determination of the potential of an amylase from any obligate halophilic fungus to be used as an additive in laundry detergent.

The molecular mass of amylases from halophilic fungi is mostly reported in the range of 50–75 kDa [25]. In this study for* A. penicillioides* TISTR 3639, it was found slightly smaller with an approximate mass of 42 kDa (Figure 1). Moreover, the specific activity of the amylase was in the moderate range [17], whilst the *K*_*m*_ value (5.41 mg·mL^−1^) of the enzyme falls in the middle of the range (0.35–11.66 mg·mL^−1^) reported for amylases from halophilic fungi [26]. The *K*_*m*_ and *V*_max⁡_ values of the enriched *α*-amylase of this study (with soluble starch as the substrate) suggested that it has a moderately high affinity for soluble starch and requires a relatively low concentration of this substrate to achieve *V*_max⁡_.

The optimal pH profile of this enriched amylase mirrors the pH-dependent growth profile of its producing* A. penicillioides*, where it was previously reported that* A. penicillioides* grew relatively well at a neutral to alkaline pH [1]. This also matches the pH found in most of the hypersaline environments [6]. The enzyme in this study retained almost 80% of its relative activity at an extreme pH (pH 11), but at pH 5 it was slightly lower at less than 75% relative activity, showing that it is potentially more tolerant of alkaline pH. An optimal pH of 9-10 has been reported for many enzymes from halophilic organisms and an alkalophilic property has been considered as the nature of halophilic enzymes [27, 28].

The alkaline amylases from microorganisms have been found to have tremendous applications in detergent industries [15]. However, most halophilic enzymes are denatured and lose their activity at temperatures over 50°C [29], whereas this halophilic and alkalophilic amylase from* A. penicillioides* TISTR 3639 showed an optimal activity at 80°C and was still relatively stable at 90°C or lower with more than 80% of its initial activity remaining after a 1 h incubation at this temperature (data not shown). Thus, it is extremely thermophilic [30, 31]. Thermophilic amylases are mostly applied in various starch industries [29].

Salinity is a crucial factor in the normal functioning of most enzymes from obligate halophilic microorganisms. Previously, it was found that* A. penicillioides* grew best at a salinity of 100 g·L^−1^ NaCl [1], a salinity level that has been widely reported to favor amylase production in most halophilic microbes [12, 32, 33]. However, the *α*-amylase from* A. penicillioides* TISTR 3639 still had a high catalytic activity even at extreme salt concentrations (300–400 g/mL^−1^ NaCl), much higher than those of the amylases reported from other extreme halophilic Archaea and Bacteria [10, 33, 34], adding a novelty to this enzyme. This extremophile property of the enzyme suggests the potential to be used in saline waste water management, in bioremediation processes in saline areas [12, 35, 36], and for biofuel production, where halophilic enzymes are reported to work better than normal enzymes [37].

The amylases from halophilic microorganisms have frequently been reported to have polyextremophilic characteristics [10, 12, 33]. They are mostly reported to be thermotolerant with haloalkalophilic properties [25], where the enzyme must have the capability to withstand extreme conditions for several industrial processes [38]. The amylase from* A. penicillioides* TISTR 3639 has the same trend of being polyextremophile, which makes it potentially versatile for use in different industrial operations, where harsh conditions are available.

The detergent industries are one of the primary consumers and users of enzymes that enhance the performance of detergents or allow the product to be more environmentally friendly [21]. Approximately 90% of commercial liquid detergents contain a mixture of enzymes that include amylases [39], since starch is considered as an attractant for various soil particles [18]. Amylases are also used in the detergent industries to remove starchy food stains, such as chocolate, custard, gravy, and potato amongst others, which are found on kitchen utensils as well as on clothes [16]. The addition of any enzyme in the detergent requires its compatibility and ability to perform in the presence of the detergents for inclusion [40]. Currently, there have been only a few reports of suitable and stable amylases that can be added into laundry detergents [41], and these need to work well at an alkaline pH [21]. Thus, it is of interest that the enriched amylase from* A. penicillioides* TISTR 3639 was found to be relatively stable in the presence of different detergents as well as alkaline conditions. Note that the compatibility test was performed under normal conditions (30°C, pH 7) that are suboptimal for this enzyme and so its activity may be improved at higher temperatures, alkalinity, and salinity levels, although of course its compatibility with these detergents would need to be evaluated at these conditions.

The rapidly changing world provides some tough challenges to humans. The limiting water resources in many parts of the world compel the use of saline or hard untreated water for daily domestic uses [42], including untreated underground hard water [43], and these decrease the cleaning efficiency of detergents [44]. In this study, the enriched *α*-amylase from* A. penicillioides* TISTR 3639, in comparison with three tested detergents and a commercial amylase, was found to work well in a low water activity, which means that the inclusion of this amylase could help solve the problem of a low cleaning capacity of detergents in a high saline environment. Moreover, when such underground hard water is not suitable for drinking it can be used for laundry purposes, saving the clean and drinkable water for consumption in areas where drinking water resources is limited.

## 5. Conclusions

Increasing demands from biotechnology, climate change, and decreasing water resources form the need to find amylases that can withstand high temperatures, salt concentrations (low water activity), and alkalinity levels. The polyextremophilic behavior of this enriched *α*-amylase obtained from* A. penicillioides* TISTR 3639 appears to be a promising candidate for fulfilling the current needs of many industrial processes requiring amylases, especially for laundry detergent industries. Of interest was the fact that the *α*-amylase from the obligate halophilic* A. penicillioides* TISTR 3639 was more extremophilic than the fungus itself. This provides an opportunity to exploit more interesting biotechnological applications from obligate halophilic fungi.

## Supplementary Material

Enzyme plate screening for extracellular α-amylase production by *A. penicillioides* TISTR 3639 on potato dextrose agar plates supplemented with 10 g.L^−1^ soluble starch. The clear zone of hydrolyzed starch was highlighted by staining the residual starch with iodine solution.

## Figures and Tables

**Table 1:** Stepwise summary of the enrichment of the *α*-amylase from *A. penicillioides* TISTR 3639.

Properties	Cell-free supernatant	(NH_4_)_2_SO_4_ precipitation	Gel filtration chromatography
Total protein (mg)	2301.1 ± 1.0	785.4 ± 1.1	219.4 ± 1.3
Total activity (U)	39142 ± 1.5 (100%)	34015.8 ± 1.0 (68%)	25891.9 ± 1.1 (44%)
Specific activity (U·mg^−1^)	17 ± 0.0	43.3 ± 0.1	118.4 ± 1.2
Purification fold	1.0	2.5	6.9

**Table 2:** Effect of various metal ions and additives on the activity of the enriched *α*-amylase from *A. penicillioides* TISTR 3639.

Additives (2 mM)	Relative activity (%)
None	100^∗f^
BaCl_2_	97.8 ± 0.8^e^
CaCl_2_	104.2 ± 1.7^g^
FeCl_2_	78.1 ± 1.5^c^
HgCl_2_	97.1 ± 1.4^e^
MgCl_2_	99.1 ± 2.2^ef^
ZnCl_2_	44.3 ± 0.9^a^
*β*-Mercaptoethanol	95.1 ± 1.5^d^
EDTA	73.4 ± 0.4^b^

^*^One hundred percent activity corresponded to the activity of the amylase without additive. Data are shown as the mean relative value ± 1SD, derived from three repeats. Means followed by a different lowercase superscript letter (a, b, c, d, e, f, and g) are significantly different (ANOVA and DMRT of the transformed data, *P* < 0.05).

**Table 3:** Effect of various detergents on the residual activity of the enriched *α*-amylase from *A. penicillioides* TISTR 3639.

Additive	Residual activity (%)
Distilled water (control)	100^∗d^
Detergent A	81 ± 1.5^a^
Detergent B	87 ± 0.1^b^
Detergent C	94 ± 1.5^c^

^*^One hundred percent activity corresponded to the activity of the amylase without any added detergent. Data are shown as the mean relative value ± 1SD, derived from three repeats. Means followed by a different lowercase superscript letter (a, b, c, and d) are significantly different (ANOVA and DMRT of the transformed data, *P* < 0.05).
