# Supplementary material for: Purification and Characterization of a Polyextremophilic α-Amylase from an Obligate Halophilic Aspergillus penicillioides Isolate and Its Potential for Souse with Detergents
Source: Biomed Res Int. 2015 Jun 9;2015:245649. doi: 10.1155/2015/245649 (PMC4477103; doi:10.1155/2015/245649)
Supplement: Supplementary file 1 — Enzyme plate screening for extracellular α-amylase production by A. penicillioides TISTR 3639 on potato dextrose agar plates supplemented with 10 g.L−1 soluble starch. The clear zone of hydrolyzed starch was highlighted by staining the residual starch with iodine solution. [file 245649.f1.pdf]

Supplementary information

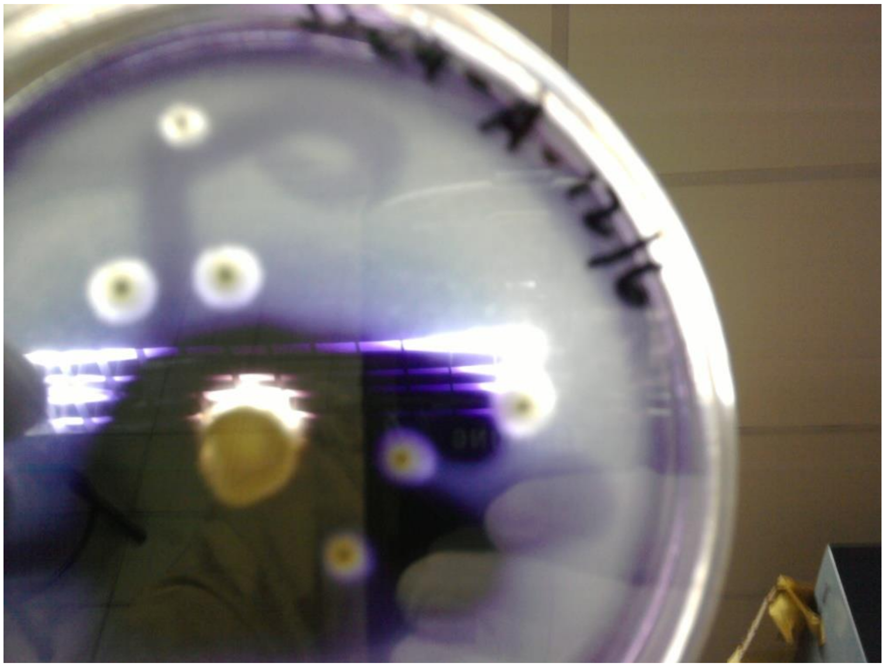

Fig. S1 Enzyme plate screening for extracellular α-amylase production by *A. penicillioides* TISTR 3639 on potato dextrose agar plates supplemented with 10 $g.L^{-1}$ soluble starch. The clear zone of hydrolyzed starch was highlighted by staining the residual starch with iodine solution.
